# Determinants of Quality of Life Among Saudi Adults with Sciatica: A Cross-Sectional Study

**DOI:** 10.3390/medicina61101824

**Published:** 2025-10-11

**Authors:** Mohammad A. Jareebi, Abdullah J. Almalki, Abdulkarim Zain Suwaydi, Ehab F. Hakami, Mnar H. Moafa, Saud N. Wadani, Fatimah N. Hakami, Shatha K. Alharbi, Malik I. Hakami, Abdulmajid E. Abuhadi, Majed A. Ryani, Ahmed A. Bahri, Yahya H. Khormi, Ibrahim A. Hakami, Abdulwahab A. Aqeeli

**Affiliations:** 1 Family and Community Medicine Department, Faculty of Medicine, Jazan University, Jazan 45142, Saudi Arabia; majedryani@gmail.com (M.A.R.); dr.bahri2010@gmail.com (A.A.B.); aaqeeli@jazanu.edu.sa (A.A.A.); 2 Faculty of Medicine, Jazan University, Jazan 45142, Saudi Arabia; abod16aj@gmail.com (A.J.A.); abdulkarimsuwaydi@gmail.com (A.Z.S.); ehab.med20@gmail.com (E.F.H.); mnarmoafa22@gmail.com (M.H.M.); saudwadani78@gmail.com (S.N.W.); fatimahnajib01@gmail.com (F.N.H.); sha.kha982001@gmail.com (S.K.A.); mr.malek360@gmail.com (M.I.H.); abdulmajidabuhadi@gmail.com (A.E.A.); 3 Department of Surgery, Faculty of Medicine, Jazan University, Jazan 45142, Saudi Arabia; khormins@gmail.com; 4 Department of Orthopedic Surgery, Dawadmi College of Medicine, Shaqra University, Shaqra 11911, Saudi Arabia; dr.ibrahim-hakami@hotmail.com

**Keywords:** sciatica, low back pain, quality of life, SF-12, Saudi Arabia, chronic pain

## Abstract

*Background and Objectives:* Sciatica is a common condition associated with significant pain and reduced quality of life (QoL). However, its impact and associated determinants remain underexplored in Saudi Arabia. Therefore, this study aimed to examine determinants of quality of life among Saudi adults with sciatica. *Materials and Methods:* A cross-sectional study was conducted in Saudi Arabia between February and March 2024 using an online Arabic questionnaire disseminated via social media. Participants (*n* = 927) aged ≥18 years completed a 50-item questionnaire covering sociodemographic characteristics, clinical history, lifestyle factors, and the Short Form-12 (SF-12) QoL scale. *Results:* Of the 927 adults (mean age 30 ± 11 years), 76% (*n* = 706) were female and 24% (*n* = 221) male; 10% (*n* = 92) reported sciatica. Overall, 57% (*n* = 531) reported good QoL. Multivariate analysis revealed that increasing age (Odds ratio (OR) = 1.04; 95% CI: 1.01–1.07), urban residence (OR = 1.49; 95% CI: 1.11–2.00), and higher income (>15,000 Saudi Arabia Riyal (SAR); OR = 1.53; 95% CI: 1.03–2.27) were associated with better QoL. Conversely, diabetes (OR = 0.42; 95% CI: 0.22–0.79), arthritis (OR = 0.52; 95% CI: 0.32–0.83), and sciatica duration >1 year (OR = 0.29; 95% CI: 0.12–0.69) were associated with poorer QoL. Gender, body mass index (BMI), smoking, and physical activity showed no significant associations. *Conclusions:* Sciatica, prolonged symptom duration, and comorbidities such as diabetes and arthritis negatively affect QoL in Saudi adults, whereas socioeconomic and demographic factors contribute positively. The results underscore the importance of early intervention and tailored support for sciatica patients with long-standing symptoms or specific comorbidities to improve health outcomes. Longitudinal studies are warranted to assess causality and the impact of interventions.

## 1. Introduction

Sciatica, characterized by radiating pain along the course of the sciatic nerve due to compression or irritation, is a prevalent and often debilitating condition that substantially impairs individuals’ physical functioning and overall quality of life (QoL) [1]. Sciatica is a clinically significant subset of low back pain (LBP), which remains the leading cause of disability worldwide and affects an estimated 577 million people globally as of 2017 [2]. The lifetime prevalence of sciatica worldwide ranges widely, from approximately 13% to 40%, with peak incidence occurring between the ages of 30 and 50 years [3,4]. This condition not only results in persistent pain but also causes activity limitation, psychological distress, and prolonged work absenteeism, placing a considerable burden on affected individuals, families, and societies [5]. Studies found that up to 30% of patients with sciatica develop some degree of disability that significantly compromises daily living activities and social participation [6].

From an economic perspective, sciatica exerts substantial pressure on healthcare systems, particularly in high-income countries, due to high healthcare utilization and indirect costs stemming from lost productivity [7]. For example, in the United States, sciatica accounts for approximately 2–5% of all primary care visits related to back pain, representing billions of dollars annually in healthcare expenditures and work-related losses [8]. Similar trends have been observed in European nations, where direct medical costs and societal losses from sciatica contribute heavily to the overall economic burden of musculoskeletal disorders [7]. Despite this considerable impact, sciatica remains underdiagnosed and undertreated in many settings, partly due to its heterogeneous clinical presentation, multifactorial pathophysiology, and variations in patient access to specialized care.

In the Middle East, and specifically in Saudi Arabia, musculoskeletal conditions such as sciatica are emerging as a major public health concern. This rise is attributable to rapid urbanization, widespread sedentary lifestyles, and high prevalence of obesity and metabolic disorders that increase vulnerability to musculoskeletal pain [9]. According to the Saudi National Health Survey, nearly 18.8% of adults report chronic low back pain, with approximately 10–12% of these cases diagnosed as sciatica [10]. However, unlike Western countries where sciatica has been extensively studied, region-specific epidemiological data on the prevalence, clinical characteristics, and QoL impact of sciatica in Saudi Arabia remain scarce. This paucity of data concerns given the distinct sociocultural and environmental factors in the region, including patterns of physical activity, occupational exposures, and healthcare utilization, which may influence disease burden and patient outcomes. Specifically, sociocultural factors such as cultural attitudes toward chronic pain, gender-specific help-seeking behaviors, and the physical demands of common occupations (e.g., construction, driving) may uniquely shape the QoL experience for Saudi sciatica patients [11].

QoL, defined broadly as an individual’s subjective perception of their physical health, psychological state, social relationships, and environment, is substantially impaired in people suffering from sciatica [12]. Multiple studies from Western contexts demonstrate that sciatica patients report significantly lower scores on validated QoL measures, including the Short Form-36 (SF-36) [13] and EuroQoL-5D (EQ-5D) [14], reflecting limitations in mobility, emotional distress, and social isolation. Chronic pain not only diminishes physical capacity but also contributes to anxiety, depression, and reduced social participation, which further degrades QoL [15]. An additional critical factor influencing QoL among sciatica patients is the duration of symptoms. Prolonged symptom duration is strongly associated with more severe functional impairment, poorer mental health outcomes, and diminished responsiveness to treatment [16].

Obesity, which a key risk factor for sciatica, is highly prevalent in Saudi Arabia, with estimates suggesting that around 35% of adults are obese [17]. This is compounded by high rates of diabetes and other metabolic conditions that may exacerbate nerve compression and inflammation, thereby worsen symptom severity and reduce QoL [18]. Early diagnosis and timely intervention are paramount to preventing chronic disability. However, evidence regarding the relationship between symptom chronicity, demographic characteristics, lifestyle factors, and QoL is limited, especially within Saudi populations [19]. Understanding these complex interactions can inform tailored interventions that address both physical and psychosocial dimensions of sciatica.

Addressing this knowledge gap, the present study aims to investigate the impact of sciatica on QoL among adults in Saudi Arabia. Specifically, this research will examine how sociodemographic variables, lifestyle behaviors, clinical factors, and symptom duration collectively influence QoL outcomes. We hypothesize that a longer duration of sciatica symptoms and the presence of obesity-related comorbidities are significantly associated with a poorer quality of life in the studied population. By providing a comprehensive assessment of these determinants, the study seeks to quantify the burden of sciatica and identify modifiable factors amenable to clinical and public health interventions.

## 2. Materials and Methods

### 2.1. Study Design, Setting, and Sampling

A cross-sectional study was conducted between February–March 2024 to assess the impact of sciatica on QoL among adults aged 18 years and above residing in the Saudi Arabia. According to the 2022 Saudi Census, the region has a population of approximately 1.5 million [20]. The minimum sample size was calculated using the formula.

$$n=\frac{\left(Z^{2}\times P\times \left(1-P\right)\right)}{d^{2}}$$

where *Z* = 1.96 for a 95% confidence level, *P* = 10% (estimated prevalence of sciatica in the region), and *d* = 3% margin of error, resulting in a minimum required sample of 384 participants [21]. Convenience sampling via online platforms was employed, an approach deemed appropriate given Saudi Arabia’s high social media penetration [22]. A total of 927 participants were recruited, substantially exceeding the minimum requirement, thereby enhancing the statistical power of the study and enabling subgroup analyses [23].

### 2.2. Study Participants and Recruitment

Eligible participants included all adults aged 18 years or older living in the Saudi Arabia who consented to participate. Recruitment was conducted via Google Forms through social media channels such as WhatsApp, Telegram, and Twitter, where the survey link was disseminated. To ensure data integrity, mandatory responses were enforced for key questions. Participants were allowed only one submission each via the Google Forms platform. Informed consent was obtained electronically before survey commencement.

### 2.3. Study Instrument and Measures

Data was collected using a structured 50-item Arabic questionnaire developed specifically for this study, based on validated instruments assessing musculoskeletal conditions and QoL [24]. To ensure content validity, the questionnaire was reviewed by a panel of three experts in public health and musculoskeletal disorders. The instrument was then pre-tested in a pilot study (*n* = 30), which demonstrated good clarity and feasibility. The internal consistency reliability of the multi-item scales was assessed using Cronbach’s alpha, which yielded a value of 0.78 for the SF-12 scale and 0.82 for the knowledge items, indicating acceptable to good reliability. Feedback from the pilot study was used to refine question wording and response options to enhance comprehension and minimize ambiguity.

The final questionnaire comprised five sections: sociodemographic characteristics (7 items), clinical history including sciatica diagnosis and duration (10 items), lifestyle factors such as physical activity and smoking (7 items), QoL assessment using the Short Form-12 (SF-12) health survey (12 items), and knowledge about sciatica symptoms and management (10 items). The 12-item Short Form Health Survey (SF-12) was used to assess participants’ QoL. The SF-12 consists of 12 questions covering eight health domains: Physical Functioning (PF), Social Functioning (SF), Role Physical (RP), Role Emotional (RE), Mental Health (ME), Vitality (VT), Bodily Pain (BP), and General Health Perception (GH) [25]. The items are aggregated, scored, and weighted to generate the Physical Component Summary (PCS) and Mental Component Summary (MCS) scores (ranging from 0, the lowest health level, to 100, the highest health level). The PCS score comprises the PF, RP, BP, and GH domains, while the MCS score comprises the SF, RE, ME, and VT domains. The SF-12 is a widely used standardized instrument designed to measure health-related QoL across two main domains: physical and mental health. It consists of 12 questions covering eight health concepts, and its scoring manual provides algorithms to compute composite physical and mental health summary scores on a scale from 0 to 100, with higher scores indicating better QoL [25]. Poor QoL was coded as the reference outcome (OR = 1). Therefore, odds ratios < 1 indicate factors associated with poorer QoL, while odds ratios > 1 indicate factors associated with better QoL.

### 2.4. Data Analysis

Data was analyzed using R software (version 4.2.3). Initial data cleaning involved checking for completeness and missing values, which were handled using Excel. Descriptive statistics (means, standard deviations, and proportions) summarized participant characteristics. Bivariate associations between sociodemographic variables, sciatica-related factors, and QoL categories were examined using Chi-square tests for categorical variables and independent *t*-tests for continuous variables. Crude odds ratios (OR) and 95% confidence intervals (CI) were calculated using univariable logistic regression with Good QoL as the reference outcome. Multiple logistic regression was conducted to identify independent predictors of QoL after controlling for potential confounders, with adjusted ORs and 95% CIs reported. Statistical significance was set at *p* < 0.05.

### 2.5. Ethical Considerations

The study protocol received ethical approval from the Jazan University Research Ethics Committee (approval number REC-45/07/973) in February 2024. Participants were fully informed about the study objectives, voluntary participation, and confidentiality prior to providing consent. Data was anonymized to ensure privacy, and no personal identifiers were collected.

### 2.6. Use of Generative Artificial Intelligence

Generative artificial intelligence (GenAI, GPT-5, OpenAI) tools were used for language editing and proofreading only. No GenAI tools were used in the design, data collection, analysis, or interpretation of the study. All content was conceived, analyzed, and interpreted by the authors.

## 3. Results

### 3.1. Sociodemographic Profile of Study Participants

Among the 927 participants (mean age 30 ± 11 years), 706 (76%) were female and 221 (24%) males. Most lived in rural areas (*n* = 519; 56%) and held undergraduate degrees (*n* = 693; 75%). The majority were single (*n* = 542; 58%), followed by married (*n* = 352; 38%). Nearly half were students (*n* = 435; 47%), while 230 (25%) worked in the government sector. Monthly family income was less than 5000 Saudi Arabia Riyal (SAR) for 319 participants (34%), 5000–9999 SAR for 182 (20%), 10,000–14,999 SAR for 175 (19%), and over 15,000 SAR for 251 (27%) (Table 1).

### 3.2. Habitual and Health-Related Characteristics

Among the 927 participants, the mean height was 159 ± 9.6 cm, weight 63 ± 17 kg, and body mass index (BMI) 25 ± 6.4 kg/m^2^. Most participants were non-smokers (*n* = 854; 92%), while 58 (6%) were current smokers and 15 (2%) were ex-smokers. Physical activity was reported by 591 participants (64%), whereas 336 (36%) were inactive. Regarding health conditions, 54 participants (6%) had diabetes and 132 (14%) reported arthritis (Table 2).

### 3.3. Sciatica Prevalence, Related Risk Factors, and Lifestyle Patterns

Out of 927 participants, 92 (10%) reported a sciatica diagnosis. In addition, 250 (27%) had a history of lower back pain accidents and 255 (28%) reported a family history of sciatica. Among all participants, 835 (90%) were never diagnosed with sciatica. Of the 92 diagnosed cases, 59 (6%) had symptoms lasting less than one year, and 33 (4%) more than one year. Regarding recovery, 76 (8%) reported incomplete recovery, 41 (4%) had recovered within the past year, 12 (1%) within 2–5 years, and 17 (2%) more than 5 years ago. Among 385 ever-married women (married, divorced, or widowed), 51 (13%) reported no pregnancies, 62 (16%) one pregnancy, 58 (15%) two pregnancies, 53 (14%) three pregnancies, and 161 (42%) more than three pregnancies. Daily phone use was <2 h for 46 (5%), 2–4 h for 232 (25%), 4–8 h for 389 (42%), and >8 h for 260 (28%). Daily driving time was <1 h for 354 (38%), 1–2 h for 365 (39%), and >2 h for 208 (22%). Overall, 531 participants (57%) reported good quality of life (QoL), while 396 (43%) reported poor QoL, with a mean score of 75 ± 20 (Table 3).

### 3.4. Bivariate Comparison of Demographic, Lifestyle, and Clinical Factors by QoL Status

Bivariate analysis revealed significant associations between multiple factors and QoL (Table 4). Increasing age was associated with better QoL (crude OR = 1.02 per year, 95% CI: 1.01–1.04, *p* < 0.001). Urban residence was associated with better QoL compared to rural residence (crude OR = 0.68 for rural, 95% CI: 0.52–0.89, *p* = 0.004). Being married was strongly associated with better QoL compared to being single (crude OR = 1.68, 95% CI: 1.27–2.22, *p* < 0.001), while student status was associated with poor QoL (crude OR = 0.57, 95% CI: 0.36–0.91, *p* = 0.019). Higher family income showed a dose–response relationship with better QoL, with the highest income category (>15,000 SAR) showing the strongest association (crude OR = 1.83, 95% CI: 1.30–2.57, *p* = 0.001).

Chronic conditions were significantly associated with poor QoL, including diabetes (crude OR = 0.53, 95% CI: 0.30–0.93, *p* = 0.027) and arthritis (crude OR = 0.66, 95% CI: 0.46–0.96, *p* = 0.028). Phone usage demonstrated a clear dose–response relationship with QoL, with longer usage associated with progressively poorer QoL. Compared to <2 h daily, usage of 4–8 h (crude OR = 0.35, 95% CI: 0.17–0.70, *p* = 0.004) and >8 h (crude OR = 0.29, 95% CI: 0.14–0.58, *p* = 0.001) were strongly associated with poor QoL.

Regarding sciatica-related factors, poor QoL was associated with sciatica diagnosis (crude OR = 0.62, 95% CI: 0.40–0.96, *p* = 0.032), history of lower back pain accidents (crude OR = 0.71, 95% CI: 0.53–0.96, *p* = 0.023), and longer symptom duration, with duration >1 year showing the strongest association (crude OR = 0.43, 95% CI: 0.23–0.80, *p* = 0.008). No significant associations were observed for gender, BMI, smoking, physical activity, education, driving duration, or family history of sciatica.

### 3.5. Multivariate Analysis of Factors Influencing QoL

**Figure 1:**
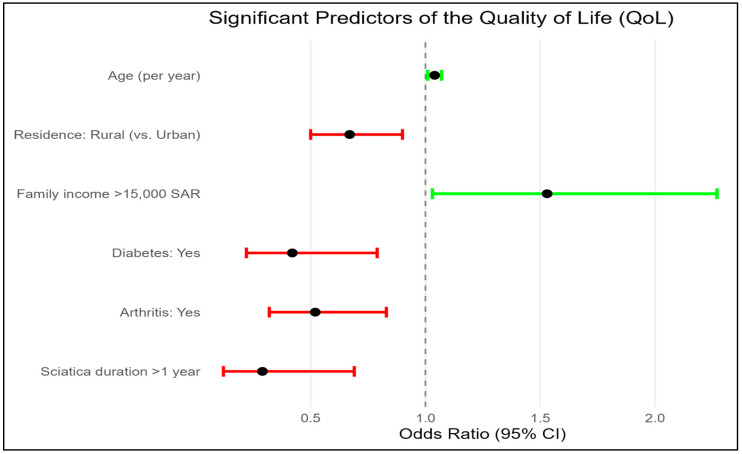
Significant predictors of quality of life (QoL) with corresponding odds ratios and 95% confidence intervals. Green indicates predictors associated with better QoL, while red indicates predictors associated with poorer QoL (*p* < 0.05).

Multiple logistic regression identified several independent predictors QoL (Table 5, Figure 1). Increasing age was significantly associated with better QoL (Odds ratios (OR) = 1.04; 95% CI: 1.01–1.07; *p* = 0.008). Higher family income was also protective, with participants earning > 15,000 SAR (>4000 USD) showing greater odds of good QoL (OR = 1.53; 95% CI: 1.03–2.27; *p* = 0.035)**.** In contrast, rural residence (OR = 0.67; 95% CI: 0.50–0.90; *p* = 0.007), diabetes (OR = 0.42; 95% CI: 0.22–0.79; *p* = 0.008), arthritis (OR = 0.52; 95% CI: 0.32–0.83; *p* = 0.007), and sciatica duration > 1 year (OR = 0.29; 95% CI: 0.12–0.69; *p* = 0.006) were significantly associated with poorer QoL. The remaining variables were not significantly associated with QoL.

## 4. Discussion

### 4.1. Key Findings Overview

This study examined demographic, health-related, and lifestyle factors influencing QoL among adults with and without sciatica in Saudi Arabia. Our findings showed that increasing age and higher income were positively associated with better QoL, while rural residence, diabetes, arthritis, and longer sciatica duration were negatively associated with QoL. Understanding these associations is crucial for clinical and community-based interventions, as it helps prioritize preventive strategies and resource allocation for at-risk populations.

### 4.2. Key Determinants of Quality of Life

Age significantly associated with better QoL, with each additional year increasing the odds of good QoL (OR = 1.04; *p* = 0.008). This may reflect psychological adaptation, coping strategies, and structural healthcare factors, consistent with studies showing higher QoL among older adults with musculoskeletal pain (mean 71.2 vs. 63.4; *p* < 0.01) [26] and better mental health scores despite higher pain (SF-36 MCS 56.1 in ≥60 years vs. 49.8 in <40 years) [27]. These findings illustrate the disability paradox, where older adults report good QoL despite chronic illness [28]. In contrast, a Dutch study reported lower QoL with age (Euro QoL 0.57 vs. 0.71 in younger adults), as this was likely because the study focused on a clinical population with more severe symptoms, and these patients also had limited social support as well as different healthcare access [29]. Unlike studies restricted to clinical settings or specific age groups, our broader adult sample provides a general perspective on age and QoL, though longitudinal studies are needed to clarify causality and reconcile inconsistent findings.

Another significant finding includes the influence of residence, where rural participants had lower odds of reporting good QoL compared to urban residents (OR = 0.67; *p* = 0.007). This disparity is likely exacerbated by gaps in Saudi Arabia’s rural healthcare infrastructure, such as fewer specialist physicians and complex referral pathways [30] and is supported by external research showing a higher prevalence of chronic low back pain in rural areas (27% vs. 18%) [31]. and significantly better urban physical functioning scores (72.4 vs. 61.3; *p* < 0.05) [32]. Beyond healthcare access, poorer outcomes may also be influenced by non-healthcare drivers, such as the higher prevalence of physically demanding rural occupations and distinct cultural health-seeking behaviors. It includes reliance on traditional remedies and community or religious healers, delayed presentation to formal healthcare, and gender- or stigma-driven patterns in seeking care. Such practices shape treatment choices, timing of care, and overall health outcomes [33,34]. However, not all studies concur; Alshami et al. found no significant difference in QoL (*p* = 0.26), a discrepancy potentially explained by their limited rural sample size (only 18% rural participants) compared to our study’s more balanced representation (urban 44% vs. rural 56%), which strengthens the reliability of our association [32]. Our study’s more balanced representation of urban (44%) and rural (56%) participants strengthens the reliability of this association. To address the barriers identified in our study, integrated interventions such as expanding telemedicine services and deploying mobile clinics could be effective solutions to bridge this healthcare gap [35].

Family income was also a key predictor of QoL, with participants earning >15,000 SAR reporting significantly higher odds of good QoL (OR = 1.53; *p* = 0.035). This exemplifies Marmot’s social gradient, where each step up the socioeconomic ladder correlates with better health [36]. In Saudi Arabia, where out-of-pocket payments constitute a significant portion of health financing and income inequality is rising (Gini coefficient ~0.45), this financial buffer is critical [37]. This suggests a protective role of financial stability, as it may facilitate better healthcare access, nutritious food, adequate housing, recreational activities, and adherence to treatment regimens through affordability and consistency of care.

This suggests a protective role of financial stability, which may enable better access to healthcare, nutritious food, better housing conditions, recreational activities, and greater adherence to treatment regimens due to affordability and consistent access to care [38,39]. This is corroborated by studies showing individuals above the poverty threshold have 1.8× higher odds of good physical functioning and 2.3× higher odds of positive mental health [40]. Similarly, low-income groups in the UK have a 36% higher risk of poor health after adjusting for age and comorbidities [41].

The presence of diabetes was significantly associated with lower QoL (OR = 0.42; *p* = 0.008), highlighting the substantial impact of chronic illness. This is consistent with studies showing individuals with diabetes have 1.5 to 2 times higher odds of poor physical functioning and greater pain interference [42,43]. Additional research found that older adults with diabetes had a 1.8-fold higher risk of reduced physical functioning and significantly lower scores on mobility and pain scales compared to non-diabetics [44]. The biological burden of neuropathic complications and fatigue directly exacerbates musculoskeletal discomfort [45,46]. Beyond pathophysiology, the constant stress of disease self-management, fear of complications, and potential stigma contribute significantly to mental health strain and reduced overall well-being [47]. This multifactorial impact is evident in longitudinal research documenting a 25% greater decline in health-related QoL over five years among diabetic patients [42]. To address this, integrated, multidisciplinary management strategies that concurrently address glycemic control, pain management, and psychological support are essential to mitigate the compounded burden on quality of life [48].

Arthritis was significantly associated with poorer QoL (OR = 0.52; *p* = 0.007), consistent with previous findings of a 1.8–2.3-fold increased disability risk and 25–40% reduced QoL scores [49,50]. Among participants with sciatica, those with comorbid arthritis (*p* < 0.01) reported significantly lower physical function scores, and this suggests a synergistic negative impact on mobility and daily activities. Such an amplification effect underscores a shared pathophysiology, as chronic inflammation and pain-related mobility restrictions combine to create a compounded burden [51]. Specifically, inflammatory markers (e.g., CRP) are elevated in both conditions and correlate strongly with pain severity (β = 0.32, *p* < 0.001) [52]. These findings highlight the critical limitation of single-disease management models. Instead, integrated musculoskeletal rehabilitation approaches combining anti-inflammatory strategies, physical therapy, and activity modification may yield superior outcomes by targeting common mechanisms rather than treating isolated diagnoses [53].

Notably, participants with sciatica lasting more than one year were significantly less likely to report good QoL (OR = 0.29; *p* = 0.006, aligning with literature showing chronic sciatica patients experience up to a 40% reduction in physical functioning [54] and a 30% decline in emotional well-being [55] compared to those with acute symptoms. While some patients demonstrate psychological adaptation over time, our findings support a trajectory of persistent disability rather than recovery, highlighting the need for early intervention. The mechanism involves ongoing nerve root irritation and chronic pain [56,57], which restricts daily activities and social participation, leading to diminished QoL. Future longitudinal studies should track pain progression and examine cultural dimensions of pain coping such as stoicism versus medicalization in Saudi populations to better understand how cultural factors influence help-seeking behavior and functional outcomes. These insights are crucial for developing culturally tailored rehabilitation programs that address both biological and psychosocial aspects of chronic sciatica.

### 4.3. Public Health Implications and Recommendations

Our findings highlight critical public health implications for sciatica management in Saudi Arabia, emphasizing modifiable risks (obesity, physical inactivity), significant rural-urban/income disparities, and comorbidity links (arthritis, diabetes). This supports implementing targeted interventions: community rehabilitation programs, workplace ergonomic initiatives, and digital health solutions (e.g., tele-rehabilitation) for rural areas.

### 4.4. Limitations

The generalizability of these findings is constrained by the cross-sectional design, a social media-based recruitment strategy that may not be representative, and a cohort predominantly comprising females (76%). Furthermore, the reliance on self-reported data for all variables, including sciatica diagnosis, introduces potential measurement inaccuracies and recall bias. These factors collectively limit causal inferences and the application of results to the broader Saudi population, particularly male demographics.

### 4.5. Future Research Directions

Future studies should adopt longitudinal designs and integrate objective diagnostic measures to clarify causal pathways. Although gender was not a significant predictor in our analysis, the predominance of female participants highlights the need for gender-sensitive approaches in future research. Additionally, diverse and representative recruitment strategies should be utilized to develop tailored, evidence-based interventions.

## 5. Conclusions

This study highlights key demographic, clinical, and lifestyle factors influencing QoL among adults with or at risk for sciatica in Saudi Arabia. Older age, urban residence, and higher income were positively associated with better QoL, while chronic conditions such as diabetes, arthritis, and prolonged sciatica significantly reduced QoL. These findings emphasize the need for targeted public health strategies and clinical interventions focused on vulnerable groups to improve overall well-being. Healthcare policymakers and providers should prioritize (1) expanding access to integrated care models that manage sciatica alongside comorbidities like diabetes and arthritis in primary care settings, and (2) investing in telemedicine and mobile health services to bridge the QoL gap for rural and low-income populations. Future longitudinal research with diverse populations and objective assessments is essential to deepen understanding and guide effective management of sciatica and related health challenges.

## Figures and Tables

**Table 1:** Sociodemographic characteristics of study participants.

Variable	*n*	Mean (SD)/Proportion (%)
**Age**	927	30 ± 11
**Gender**	927	
Female	706	76%
Male	221	24%
**Residence**	927	
Urban	408	44%
Rural	519	56%
**Education**	927	
Postgraduate	60	6%
Secondary school or less	174	19%
Undergraduate	693	75%
**Marital status**	927	
Married	352	38%
Single	542	58%
Widowed/Divorced	33	4%
**Employment**	927	
Employed in governmental sector	230	25%
Employed in private sector	49	5%
Housewife	93	10%
Retired	29	3%
Student	435	47%
Unemployed	91	10%
Family income *	927	
Less than 5000 SAR (<1333 USD)	319	34%
5000–9999 SAR (1333–2666 USD)	182	20%
10,000–14,999 SAR (2667–3999 USD)	175	19%
More than 15,000 SAR (>4000 USD)	251	27%

* 1 Saudi Riyal (SAR) ≈ 0.27 USD.

**Table 2:** Habitual and health related characteristics.

Variable	*n*	Mean (SD)/Proportion (%)
**Height**	927	159 ± 9.6
**Weight**	927	63 ± 17
**Body mass index (BMI)**	927	25 ± 6.4
**Smoking**	927	
No	854	92%
Ex-smoker	15	2%
Yes	58	6%
**Physical Activity**	927	
No	336	36%
Yes	591	64%
**Diabetes**	927	
No	873	94%
Yes	54	6%
**Arthritis**	927	
No	795	86%
Yes	132	14%

**Table 3:** Sciatica related characteristics and risk factors.

Variable	*n*	Mean (SD)/Proportion (%)
**Sciatica Diagnosis**	927	
No	835	90%
Yes	92	10%
**Lower back pain (LBP)**	927	
No	677	73%
Yes	250	27%
**Sciatica Family**	927	
No	672	72%
Yes	255	28%
**Sciatica Duration**	927	
Less Than One Year	59	6%
More Than One Year	33	4%
Never Diagnosed with Sciatica	835	90%
**Sciatica Recovery**	927	
No	76	8%
Yes, A Year Ago	41	4%
Yes, For 2–5 Years	12	1%
Yes, Since More Than 5 Years	17	2%
Never Diagnosed with Sciatica	835	90%
**Pregnancy** (married, divorced and widowed)	385	
0	51	6%
1	62	7%
2	58	6%
3	53	6%
More Than 3 Times	161	17%
**Phone Usage**	927	
2–4 h	232	25%
4–8 h	389	42%
Less Than 2 h	46	5%
More Than 8 h	260	28%
**Driving per day**	927	
1–2 h	365	39%
Less Than 1 h	354	38%
More Than 2 h	208	22%
**QoL**	927	
Good	531	57%
Poor	396	43%
**QoL Score**	927	75 ± 20

**Table 4:** Bivariate analysis of factors associated with quality of life **.

Variable	Good QoL (*n* = 531)	Poor QoL (*n* = 396)	Crude OR	95% CI	*p*-Value *
**Age (mean ± SD)**	31 ± 12	28 ± 11	1.02	1.01–1.04	<0.001 ***
**Gender**					0.105
Female	394 (74%)	312 (79%)	Ref	-	
Male	137 (26%)	84 (21%)	1.29	0.95–1.76	
**Height (mean ± SD)**	160 ± 9.3	159 ± 10	1.01	0.99–1.02	0.314
**Weight (mean ± SD)**	63 ± 17	62 ± 18	1.00	1.00–1.01	0.243
**BMI (mean ± SD)**	25 ± 6.2	24 ± 6.6	1.01	0.99–1.03	0.414
**Residence**					0.004 **
Urban	255 (48%)	153 (39%)	Ref	-	
Rural	276 (52%)	243 (61%)	0.68	0.52–0.89	
**Education**					0.293
Secondary school or less	93 (18%)	81 (20%)	Ref	-	
Undergraduate	401 (76%)	292 (74%)	1.20	0.86–1.67	
Postgraduate	37 (7%)	23 (6%)	1.40	0.77–2.58	
**Marital status**					<0.001 ***
Single	285 (54%)	257 (65%)	Ref	-	
Married	229 (43%)	123 (31%)	1.68	1.27–2.22	
Widowed/Divorced	17 (3%)	16 (4%)	0.96	0.47–1.95	
**Employment**					0.019 *
Unemployed	58 (11%)	33 (8%)	Ref	-	
Government sector	143 (27%)	87 (22%)	0.94	0.56–1.54	
Private sector	32 (6%)	17 (4%)	1.07	0.52–2.24	
Housewife	55 (10%)	38 (10%)	0.82	0.45–1.49	
Retired	22 (4%)	7 (2%)	2.42	0.81–8.94	
Student	218 (41%)	217 (55%)	0.57	0.36–0.91	
**Family income**					0.001 ***
<5000 SAR	162 (31%)	157 (40%)	Ref	-	
5000–9999 SAR	109 (21%)	73 (18%)	1.45	1.00–2.10	
10,000–14,999 SAR	96 (18%)	79 (20%)	1.18	0.81–1.71	
>15,000 SAR	164 (31%)	87 (22%)	1.83	1.30–2.57	
**Smoking**					0.590
No	487 (92%)	367 (93%)	Ref	-	
Yes	44 (8%)	29 (7%)	1.14	0.71–1.88	
**Physical activity**					0.949
No	192 (36%)	144 (36%)	Ref	-	
Yes	339 (64%)	252 (64%)	1.01	0.77–1.32	
**Diabetes**					0.027 *
No	508 (96%)	365 (92%)	Ref	-	
Yes	23 (4%)	31 (8%)	0.53	0.30–0.93	
**Arthritis**					0.028 *
No	467 (88%)	328 (83%)	Ref	-	
Yes	64 (12%)	68 (17%)	0.66	0.46–0.96	
**Phone usage**					0.001 ***
<2 h	35 (7%)	11 (3%)	Ref	-	
2–4 h	165 (31%)	67 (17%)	0.77	0.36–1.57	
4–8 h	206 (39%)	183 (46%)	0.35	0.17–0.70	
>8 h	125 (24%)	135 (34%)	0.29	0.14–0.58	
**Car driving per day**					0.432
<1 h	201 (38%)	153 (39%)	Ref	-	
1–2 h	219 (41%)	146 (37%)	1.14	0.85–1.54	
>2 h	111 (21%)	97 (24%)	0.87	0.62–1.23	
**Sciatica diagnosis**					0.032 *
No	488 (92%)	347 (88%)	Ref	-	
Yes	43 (8%)	49 (12%)	0.62	0.40–0.96	
**History of LBP accident**					0.023 *
No	403 (76%)	274 (69%)	Ref	-	
Yes	128 (24%)	122 (31%)	0.71	0.53–0.96	
**Family history of sciatica**					0.066
No	396 (75%)	276 (70%)	Ref	-	
Yes	135 (25%)	120 (30%)	0.78	0.59–1.04	
**Sciatica duration**					0.008 **
Never diagnosed	488 (92%)	347 (88%)	Ref	-	
<1 year	26 (5%)	33 (8%)	0.64	0.41–0.99	
>1 year	17 (3%)	27 (7%)	0.43	0.23–0.80	

* Statistical significance markers * *p* < 0.05; ** *p* < 0.01; *** *p* < 0.001. ** Crude odds ratios calculated using Good QoL as the reference outcome. OR < 1 indicates factors associated with poor QoL; OR > 1 indicates factors associated with good QoL.

**Table 5:** Multiple logistic regression of the association between sciatica and QoL (*n* = 972).

	QoL		
Predictors	OR	95% CI	*p*-Value
(Intercept)	1.11	0.39–3.16	0.841
**Age**	1.04	1.01–1.07	0.008 **
**Gender** (Ref: Female)			
Male	1.23	0.82–1.84	0.321
**BMI**	0.99	0.96–1.01	0.312
**Residence** (Ref: Urban)	0.67	0.50–0.90	0.007
Rural			
**Education** (Ref: High school or less)			
Postgraduate	0.82	0.41–1.67	0.589
Undergraduate	1.11	0.77–1.61	0.575
**Marital status** (Ref: Single)			
Married	1.22	0.70–2.15	0.488
Widowed/Divorced	0.73	0.30–1.77	0.482
**Employment** (Ref: Unemployed)			
Governmental sector	0.66	0.32–1.33	0.242
Private sector	0.87	0.40–1.92	0.729
Housewife	0.71	0.35–1.46	0.355
Retired	1.48	0.37–6.85	0.589
Student	0.64	0.38–1.06	0.088
**Family income** (Ref: Less than 5000 SAR)			
From 10,000 to 14,999 SAR	1.05	0.68–1.62	0.816
From 5000 to 9999 SAR	1.33	0.89–2.00	0.164
More than 15,000 SAR	1.53	1.03–2.27	0.035
**Smoking** (Ref: No)			
Yes	0.84	0.47–1.52	0.563
**Physical Activity** (Ref: No)			
Yes	0.94	0.70–1.26	0.673
**Diabetes** (Ref: No)			
Yes	0.42	0.22–0.79	0.008 **
**Arthritis** (Ref: No)			
Yes	0.52	0.32–0.83	0.007 **
**Sciatica diagnosis** (Ref: No)			
Yes	1.36	0.64–2.94	0.432
**Sciatica family** (Ref: No)			
Yes	0.86	0.62–1.21	0.388
**Lower Back Pain** (Ref: No)			
Yes	0.73	0.53–1.02	0.061
**Sciatica duration**			
Less than one year	0.52	0.26–1.03	0.063
More than one year	0.29	0.12–0.69	0.006 **

Statistical significance marker ** *p* < 0.01.

## Data Availability

The data supporting the findings of this study are available from the corresponding author upon reasonable request.

## Abbreviations

The following abbreviations are used in this manuscript:

QoL	Quality of Life
SF-12	Short Form-12 Health Survey
LBP	Low Back Pain
SAR	Saudi Riyal
OR	Odds Ratio
CI	Confidence Interval
